# P-1073. Clinical, microbiological characteristics and predictors of adverse outcomes in Chryseobacterium spp. bloodstream infections

**DOI:** 10.1093/ofid/ofaf695.1268

**Published:** 2026-01-11

**Authors:** Jinghao Nicholas Ngiam, Matthew CY Koh, David M Allen, Ka Lip Chew

**Affiliations:** National University Health System, Singapore, Singapore; National University Health System, Singapore, Singapore; NUHS, Singapore, Not Applicable, Singapore; National University Hospital, Singapore, Singapore, Not Applicable, Singapore

## Abstract

**Background:**

*Chryseobacterium* spp. are non-fermentative, Gram-negative bacilli that are intrinsically carbapenem resistant. They are typically environmental organisms but can cause nosocomial infections, particularly in relation to indwelling medical devices. Optimal antimicrobial therapy for this relatively rare infection remains unclear. We aimed to evaluate the clinical and microbiological characteristics, as well as identify predictors of mortality amongst bloodstream infections with this organism.

**Table 1:**
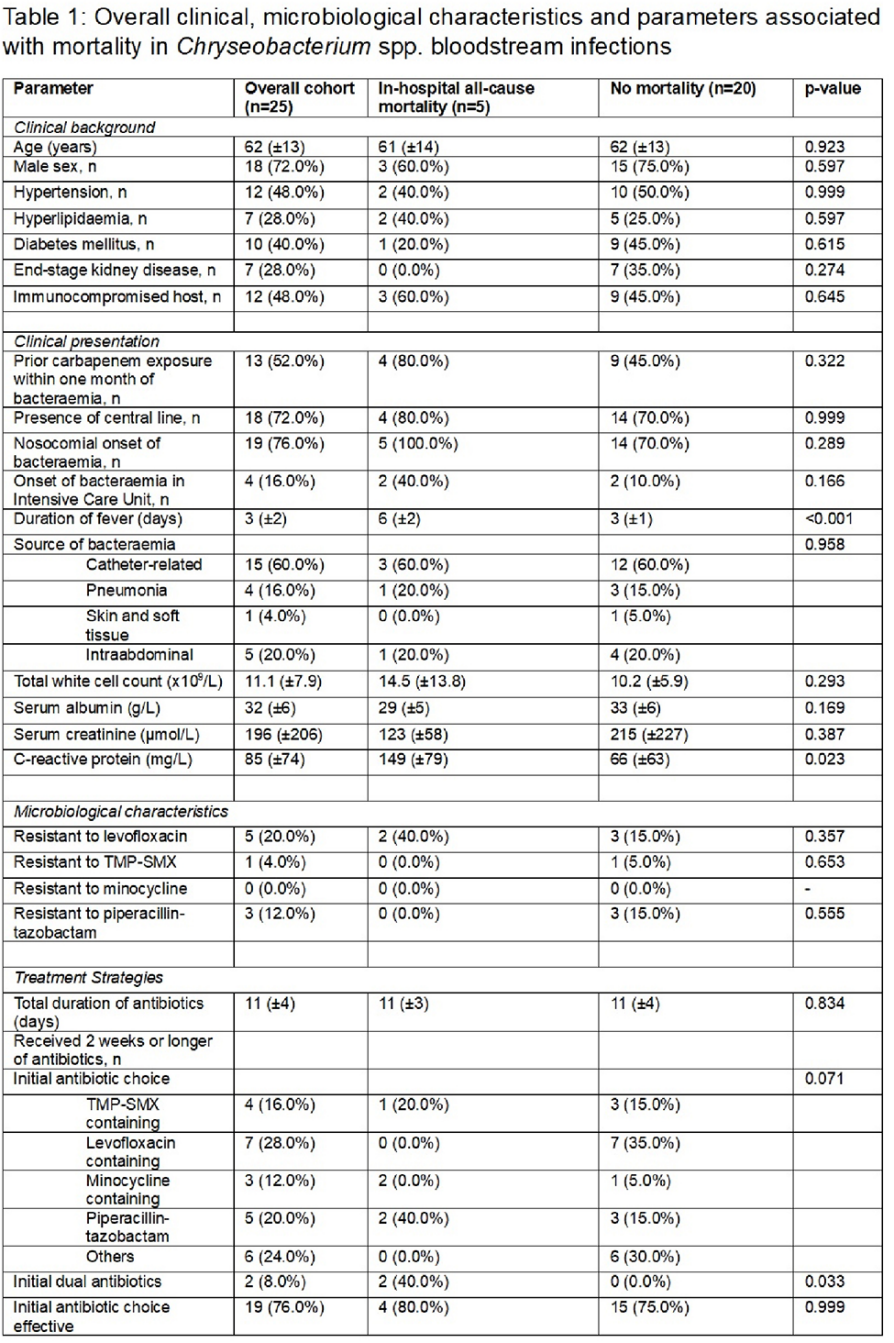
Overall clinical, microbiological characteristics and parameters associated with mortality in Chryseobacterium spp. bloodstream infections

**Methods:**

We retrospectively examined consecutive patients with *Chryseobacterium* spp. bacteraemia from 2012-2024 from a single tertiary institution in Singapore. These patients were divided into those who experienced in-hospital mortality, and those who did not. We compared the clinical and microbiological characteristics between these groups to identify predictors of the adverse outcome.

**Results:**

Amongst the infections with *Chryseobacterium* spp., 40% were speciated as *C. indologenes,* and 30% were *C. gleum*. A majority of these infections were nosocomial in onset (76.0%). The commonest foci of infection were catheter-related (60.0%), intra-abdominal (20.0.%) and pneumonia (16.0%). In terms of microbiology, 20% of the isolates were resistant to levofloxacin, and 12% were resistant to piperacillin-tazobactam. Only one isolate (4.0%) was resistant to trimethoprim-sulfamethoxazole, and all isolates were susceptible to minocycline. In-hospital mortality was observed in 20% (5/25) patients. A longer duration of fever (6±2 vs 3±1 days, p< 0.001) and elevated C-reactive protein (149±79 vs 66±63 mg/L, p=0.023) were associated with mortality.

**Conclusion:**

*Chryseobacterium* spp. may be a rare but important nosocomial pathogen, with significant morbidity and mortality. Understanding antimicrobial susceptibility patterns may help guide empiric antibiotic choices. Prolonged fever and elevated C-reactive protein appear to be poor prognostic markers.

**Disclosures:**

All Authors: No reported disclosures

